# Supplementary material for: Cryo-EM structures of lipidic fibrils of amyloid-β (1-40)
Source: Nat Commun. 2024 Feb 13;15:1297. doi: 10.1038/s41467-023-43822-x (PMC10864299; doi:10.1038/s41467-023-43822-x)
Supplement: Supplementary file 1 — Supplementary Information [file 41467_2023_43822_MOESM1_ESM.pdf]

Supplementary Information

Cryo-EM structures of lipidic fibrils of amyloid-β(1-40)

Benedikt Frieg[1#], Mookyoung Han[2#], Karin Giller[2], Christian Dienemann[3], Dietmar Riedel[6] Stefan Becker[2*], Loren B. Andreas[2*], Christian Griesinger[2,4*], and Gunnar F. Schröder[1,5*]

[1] Institute of Biological Information Processing (IBI-7: Structural Biochemistry) and JuStruct: Jülich Center for Structural Biology, Forschungszentrum Jülich; Jülich, Germany.

[2] Department of NMR-Based Structural Biology, Max Planck Institute for Multidisciplinary Sciences; Göttingen, Germany.

[3] Department of Molecular Biology, Max Planck Institute for Multidisciplinary Sciences; Göttingen, Germany.

[4] Cluster of Excellence "Multiscale Bioimaging: From Molecular Machines to Networks of Excitable Cells" (MBExC), University of Göttingen; Göttingen, Germany.

[5] Physics Department, Heinrich Heine University Düsseldorf; Düsseldorf, Germany

[6]Laboratory of Electron Microscopy, Max-Planck-Institute for Multidisciplinary Sciences, Göttingen, Germany.

# These authors contributed equally to this work.

* Correspondence and requests for materials should be addressed to Gunnar F. Schröder (gu.schroeder@fz-juelich.de), Christian Griesinger (cigr@mpinat.mpg.de), Loren B. Andreas (land@mpinat.mpg.de), or Stefan Becker (sabe@mpinat.mpg.de).

Supplementary Figures

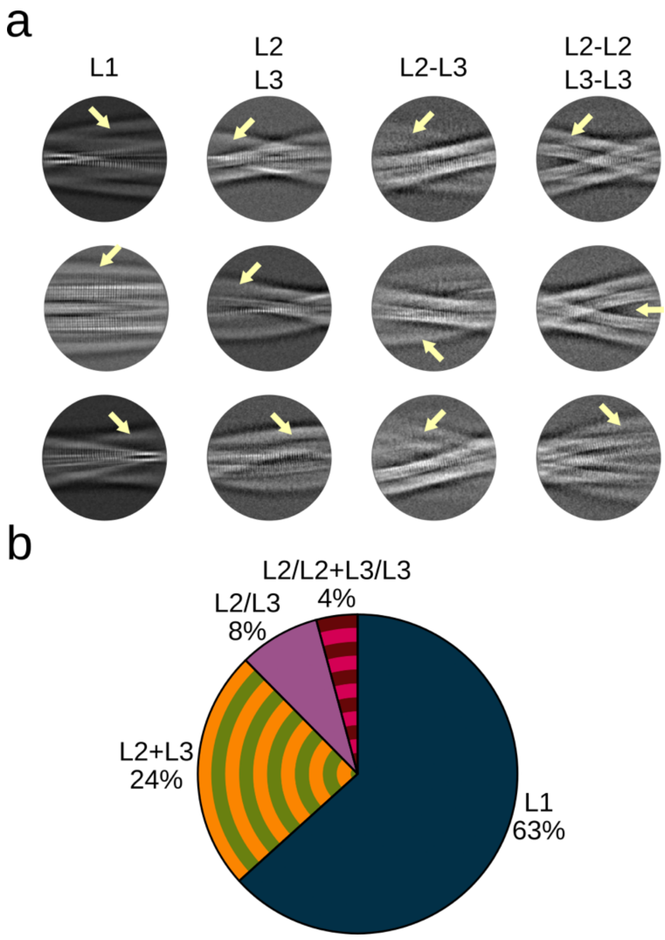

Supplementary Figure 1 | Cryo-EM of lipidic Aβ40 fibrils.

a, A 20 Å low-pass filtered cryo-electron microscopy (cryo-EM) micrograph of lipidic Aβ40 fibrils. Examples of 2D class averages for all structural polymorphs. Due to structural similarities, *L2* and *L3* as well as *L2-L2* and *L3-L3* fibrils are grouped. The arrows indicate the fibril-bound layers of lipids, lacking the characteristic amyloid cross-β pattern, while the Aβ40 fibrils can be identified by the cross-β pattern. **b,** Pie chart visualizing the relative population (in %) of each lipidic Aβ40 fibril after 2D classification (based on results shown in **a**). As L2 and L3 fibrils and L2/L2 and L3/L3 were separated after multiple rounds of 3D classification, individual estimates are not available.

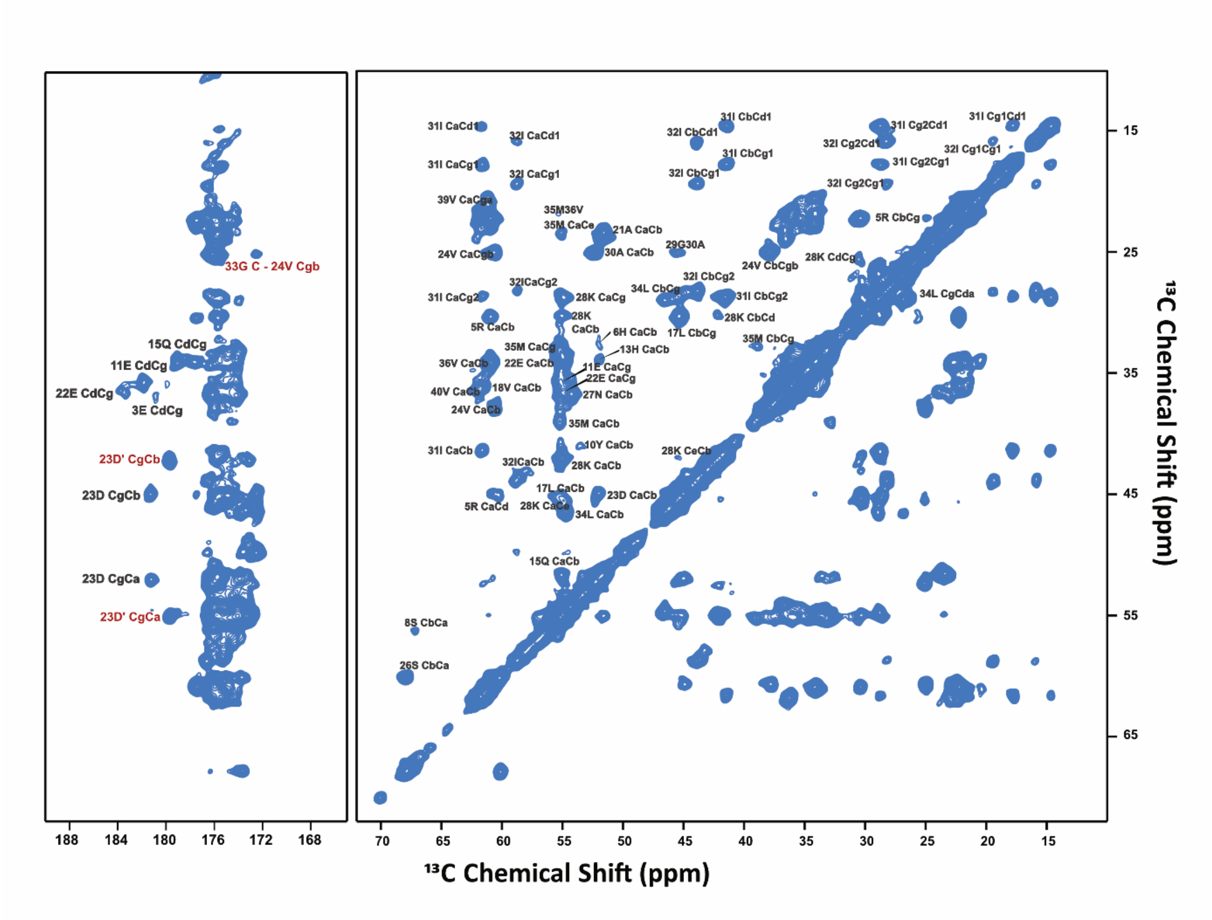

Supplementary Figure 2 | Backbone and side-chain spectrum of Aβ40 fibrils in the presence of phospholipids.

Carbonyl and aliphatic region (50ms) of the 2D ^{13}C-^{13}C chemical shift correlation spectrum of the $^{13}C^{15}N$-labeled lipidic Aβ40 fibril.

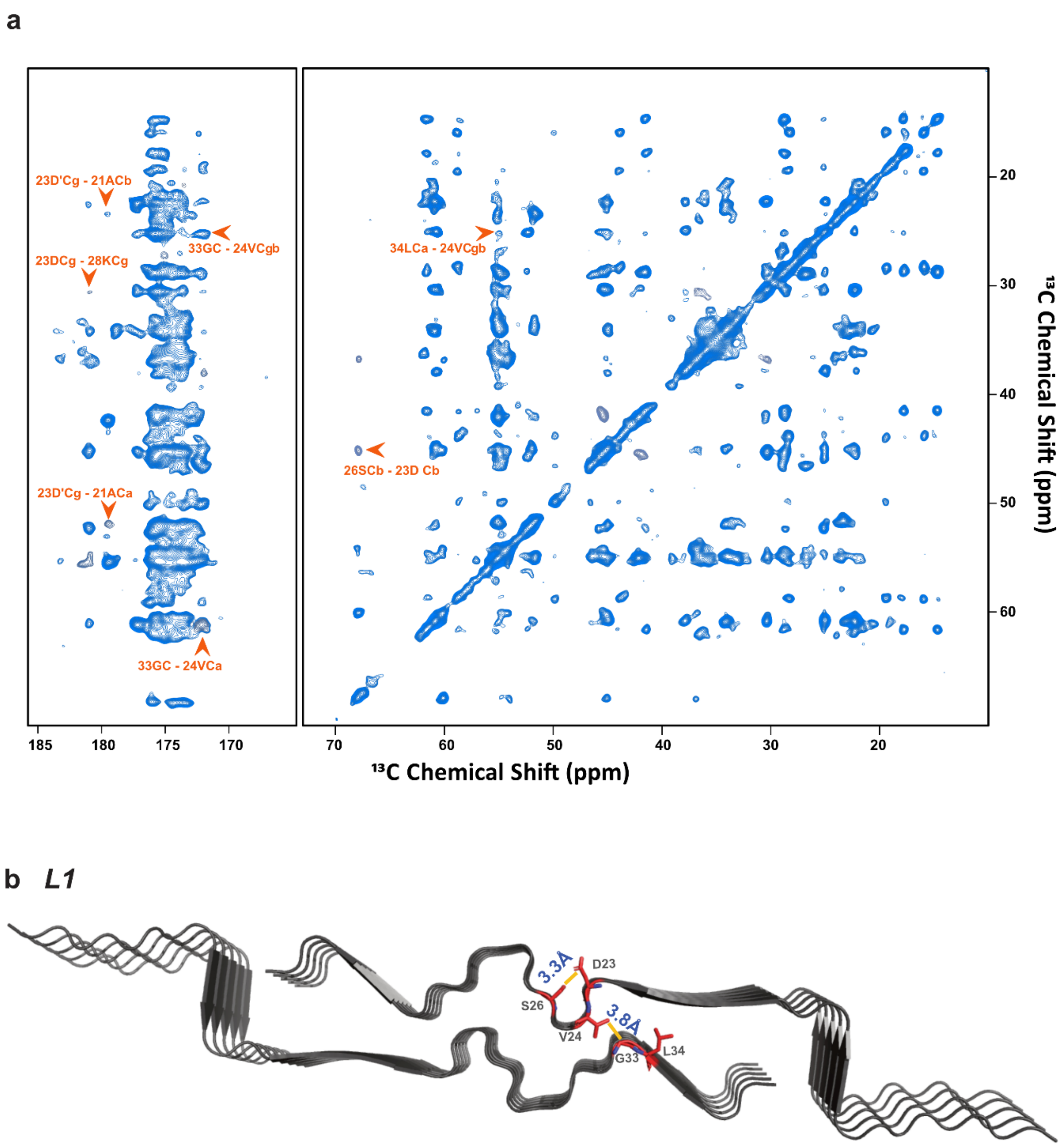

Supplementary Figure 3 | Cross contact between the protofilaments.

a, 2D ^{13}C-^{13}C chemical shift correlation spectrum (200ms) of the $^{13}C^{15}N$ lipidic Aβ40 fibril. Orange arrows indicate the middle range (2 Å ~ 5 Å) contact between the two residues. **b,** The structure of L1. Yellow bars indicate the distance between two residues.

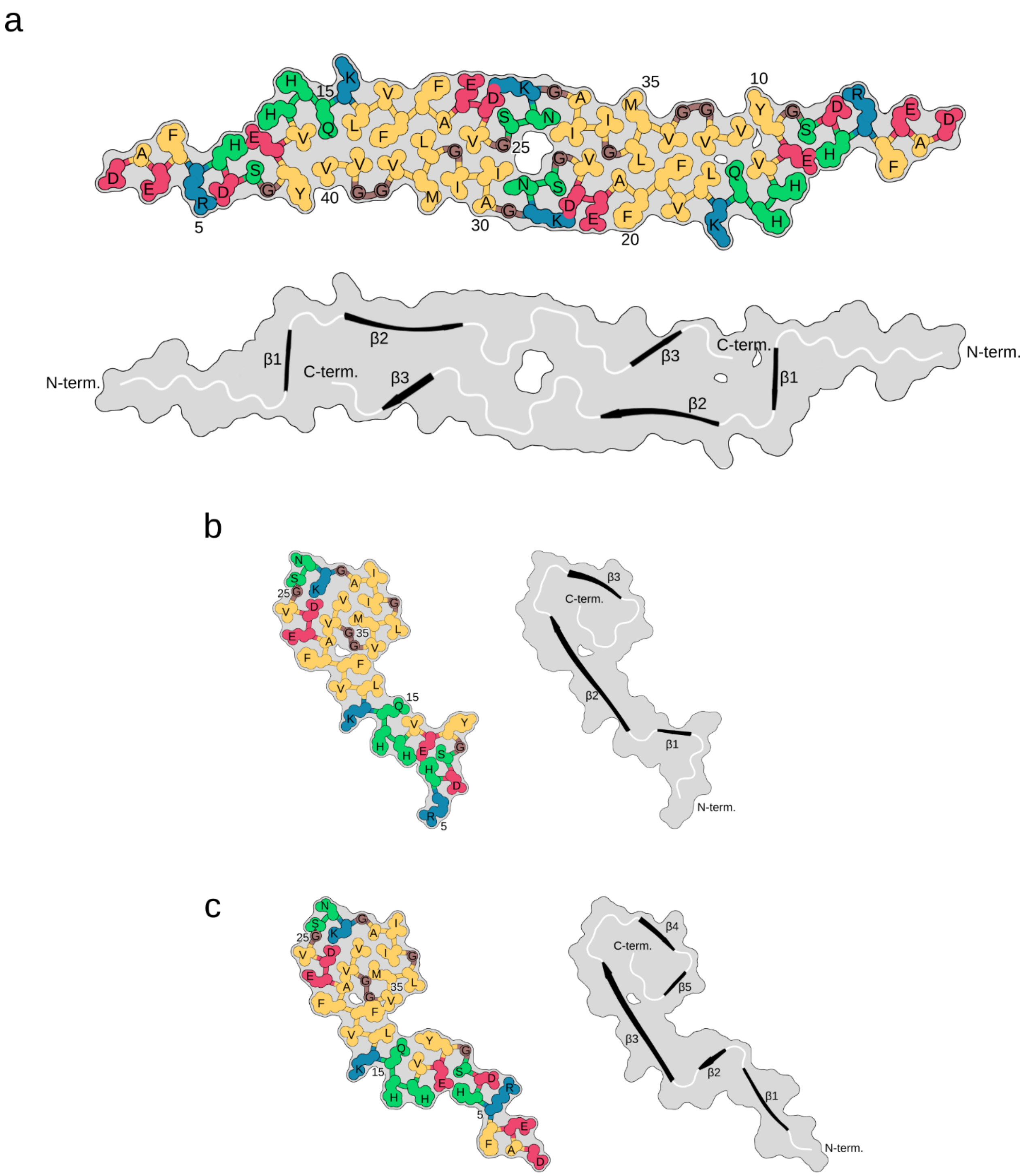

Supplementary Figure 4 | Distribution and properties of residues in the lipidic *L1*, *L2*, and *L3* Aβ folds.

One panel shows polar residues in green, apolar residues in yellow, negatively charged in red, positively charged in blue, and glycine in brown for the *L1* (**a**), *L2* (**b**), and *L3* (**c**) fibril. The other panel shows β-strands (β1 - β4) in black and loops in white.

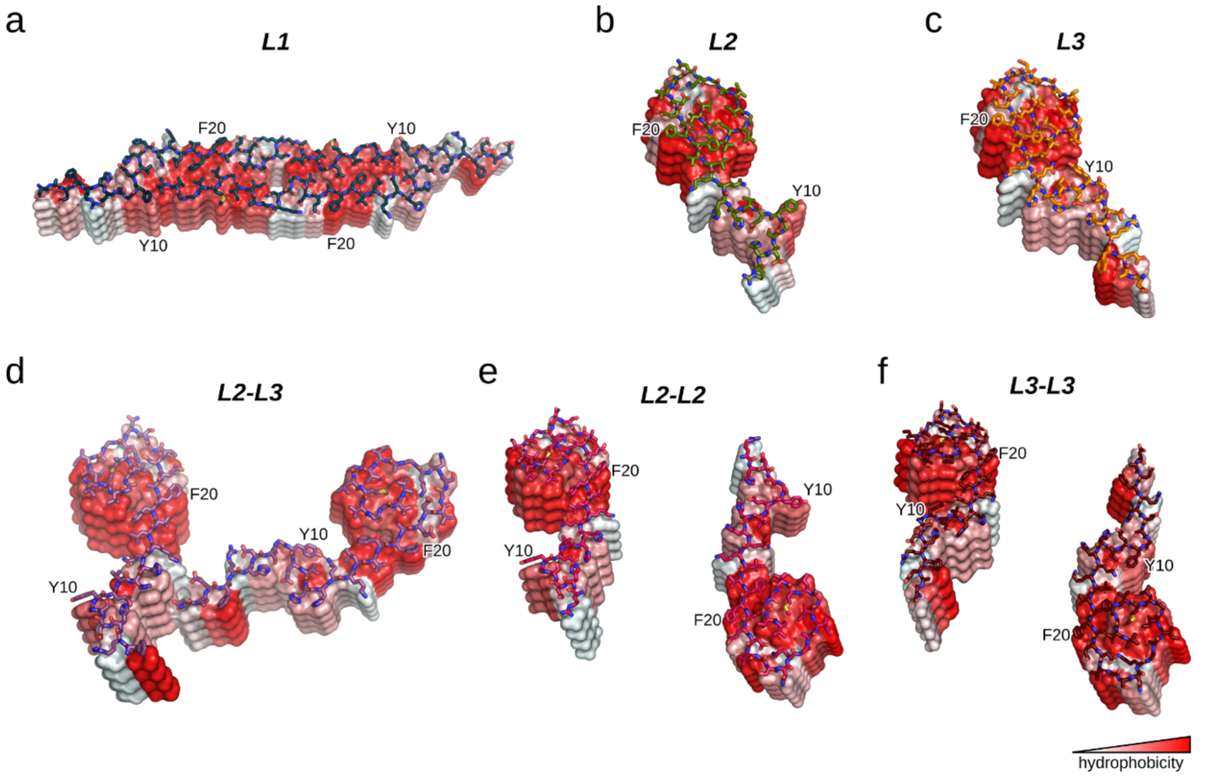

Supplementary Figure 5 | Surface properties of the lipidic Aβ40 fibrils.

Surface representation of the *L1* (**a**), *L2* (**b**), *L3* (**c**), *L2-L3* (**d**), *L2-L2* (**e**), and *L3-L3* (**f**) fibrils. The surface is colored according to the Eisenberg hydrophobicity scale[67]. The top layer is shown as a stick model. In all fibrils, Y10 and F20 belong to hydrophobic patches, which are essential for fibril-lipid interactions.

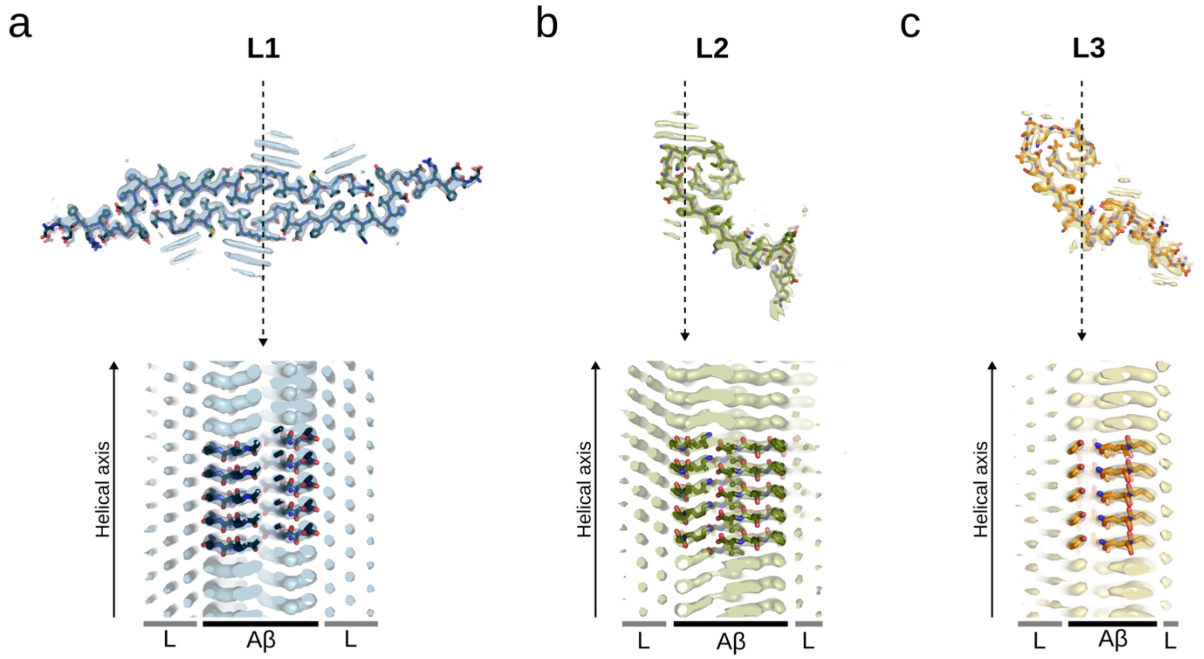

Supplementary Figure 6 | Cryo-EM structures of lipidic Aβ40 fibrils.

Cross-sections of *L1* (**a**), *L2* (**b**), and *L3* (**c**) folds. The bottom panels show cross-sections of the fibrils in side-view (10 Å around the dash-lined arrow), visualizing the periodical arrangement of the rod-shaped lipid densities (L) along the helical axis seen in the cryo-EM maps.

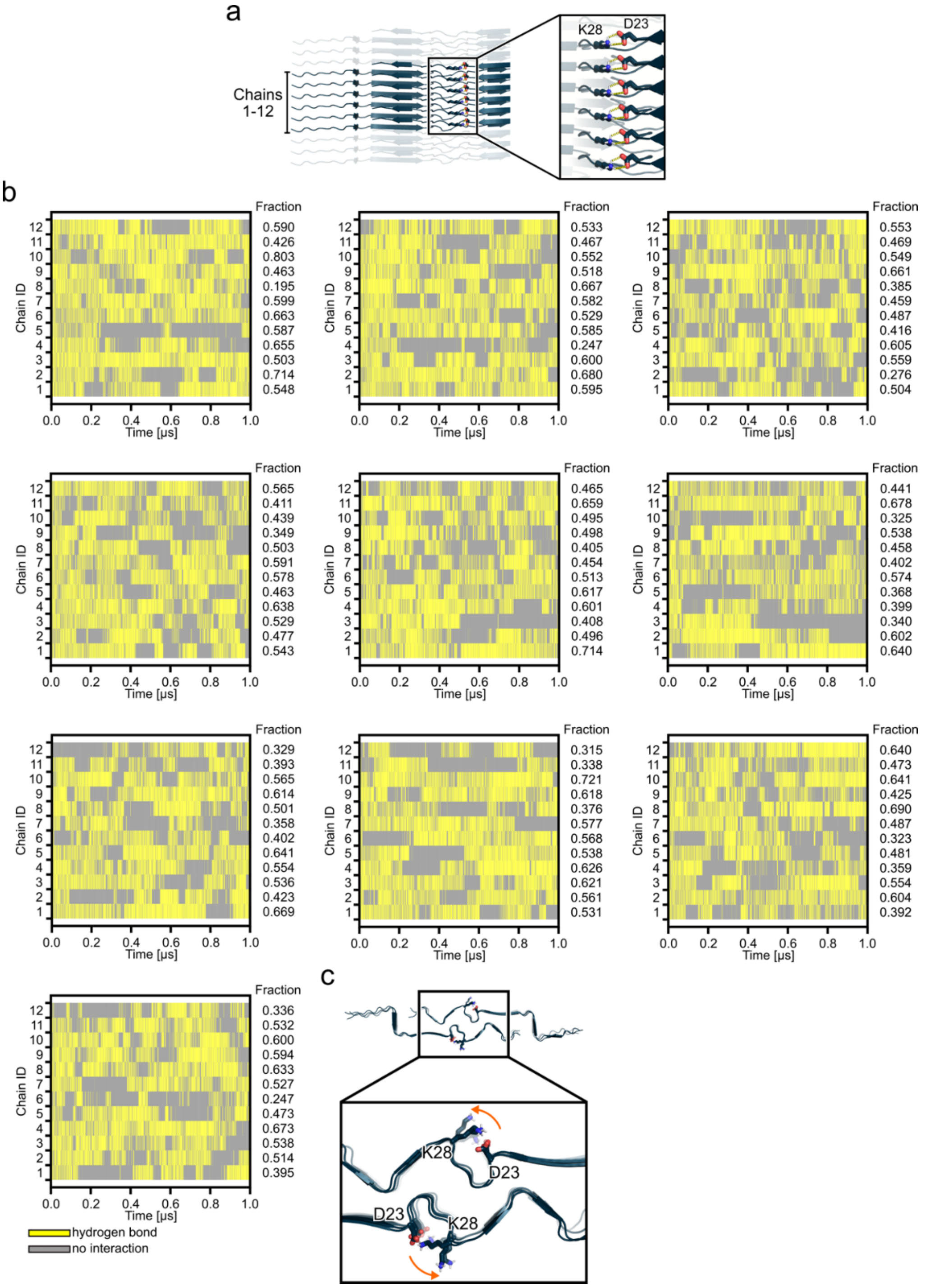

Supplementary Figure 7 | Analysis of the intra-molecular hydrogen bond between D23 and K28 during MD simulations.

a, The atomic model of the lipidic L1 Aβ40 40 fibril is shown, which served as the input structure for MD simulations. For trajectory analysis, we only considered the central 12 chains. The close-up view shows the intra-molecular hydrogen bond (shown as dashed lines) between D23 and K28 (shown as stick models). **b**, Hydrogen bond analysis during 10 x 1.0 µs MD simulations of the lipidic L1 Aβ40 40 fibril for the central 12 Aβ40 40 chains. Yellow regions denote conformations in which the D23-K28 hydrogen bond is present, while any interaction

is absent in gray regions. The fraction reports how many conformations the D23-K28 hydrogen bond is found. **c**, Representative structure after 1.0 μs MD simulations. The arrow visualizes the side-chain movement of K28, resulting in a loss of the hydrogen bond interactions with D23.

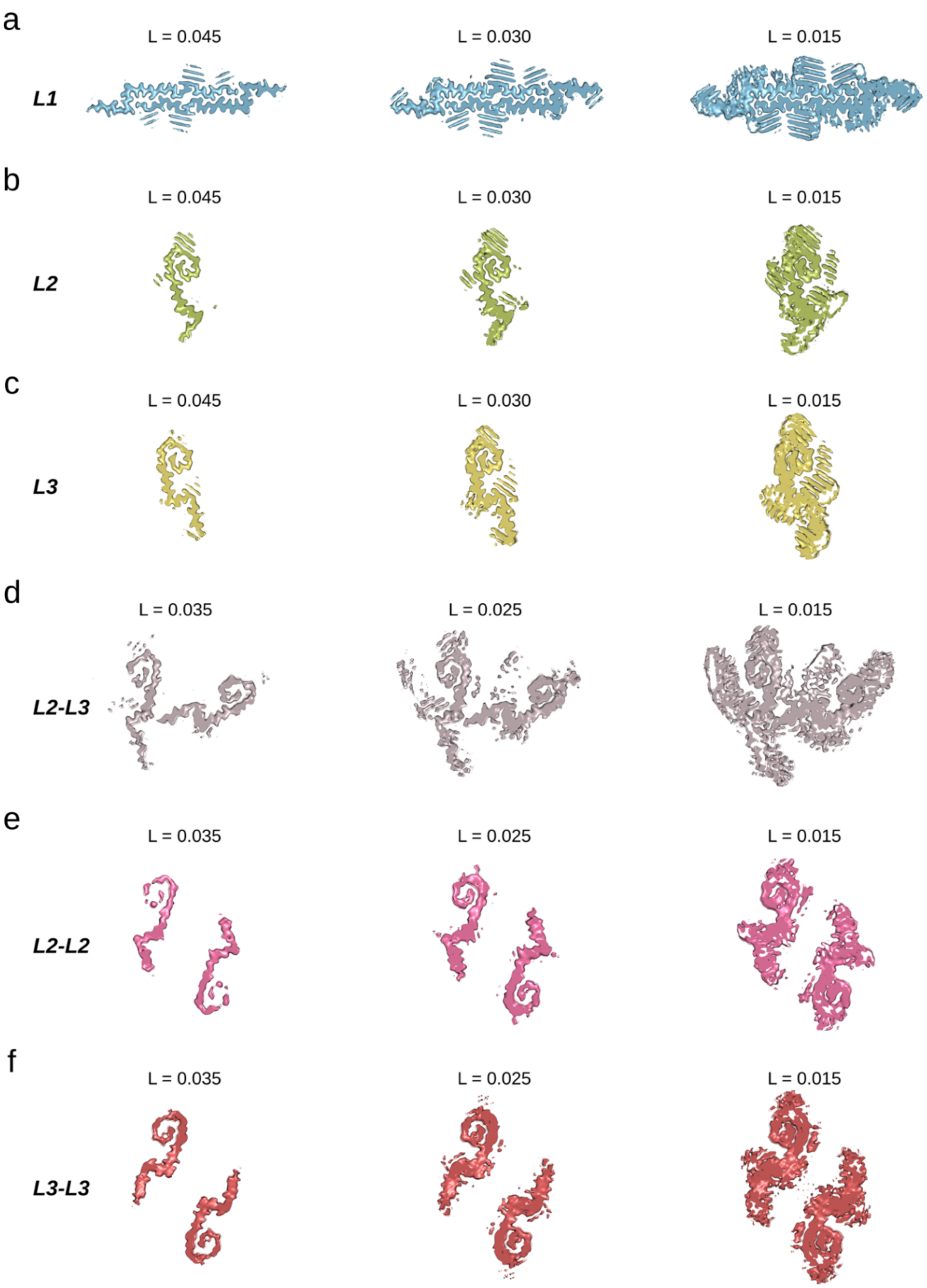

100 **Supplementary Figure 8 | Cryo-EM maps at different contour levels.**
Cross-sections of the density maps shown at different contour level thresholds (L) for the *L1* (**a**, blue), *L2* (**b**, green), *L3* (**c**, orange), *L2-L3* (**d**, purple), *L2-L2* (**e**, magenta), and *L3-L3* (**f**, red) fibrils.

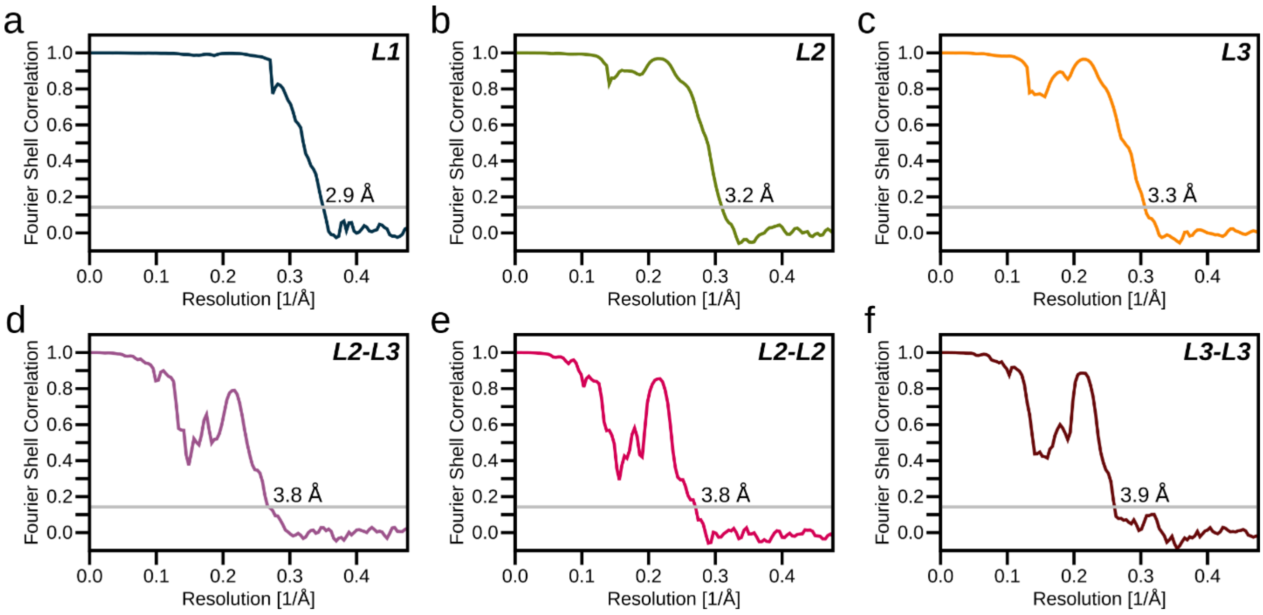

Supplementary Figure 9 | Fourier shell correlation curves.

Mask-corrected (z-percentage is 0.1) Fourier shell correlation (FSC) curves for *L1* (**a**), *L2* (**b**), *L3* (**c**), *L2-L3* (**d**), *L2-L2* (**e**), and *L3-L3* (**f**). The plots show the final resolutions, which were estimated from the value of the FSC curve for two separately refined masked half-maps at 0.143 (gray line).

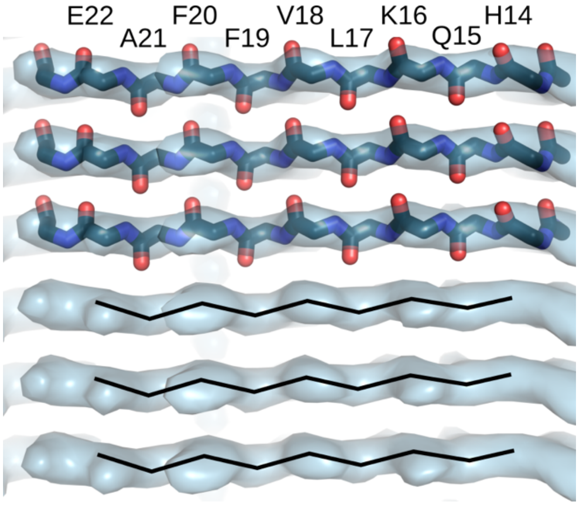

Supplementary Figure 10 | Lateral view the β-sheet formed by residues H14 to E22 in the L1 fibril.

Overlay of the final cryo-EM map (surface) and the backbone model of residues H14 to E22 (sticks). The black lines show the backbone carbonyl directions, suggesting a left-handed helical symmetry.

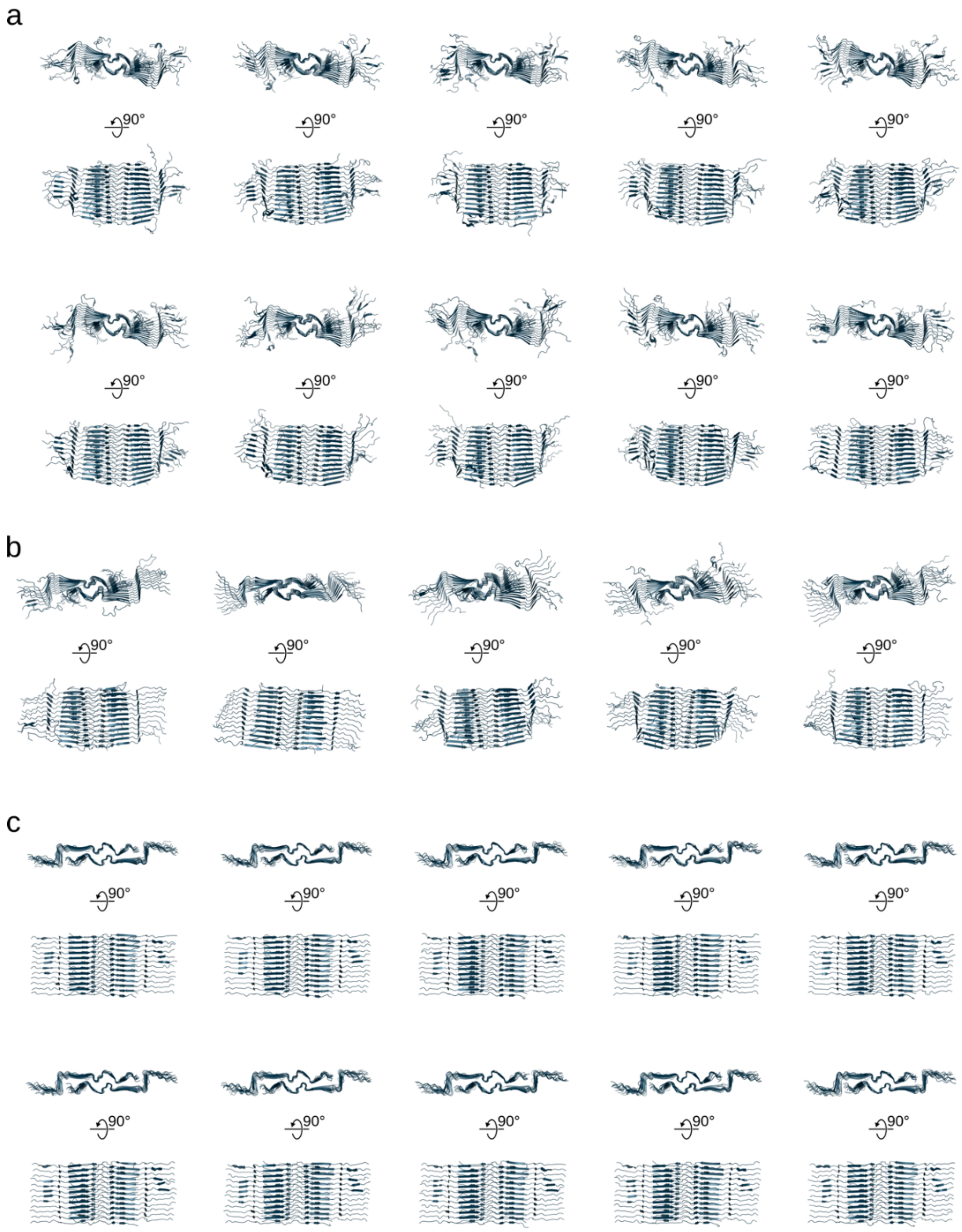

Supplementary Figure 11 | Molecular dynamics simulations of the L1 fibril.
a, Conformations of the L1 fibril extracted from ten molecular dynamics trajectories after 1 µs without restraints. **b**, Representative conformations of the five highest populated clusters, considering all conformations from ten molecular dynamics trajectories of 1 µs length without restraints. Without the final proper arrangement of lipids around the fibril and without additional restraints, the N- and C-termini as well as the top and bottom stacked protein layers are highly mobile. **c**, Conformations of the L1 fibril extracted from ten molecular dynamics trajectories after 1 µs with additional weak restraints (0.1 kcal mol^{-1} $Å^{-2}$) on the Cα atoms.

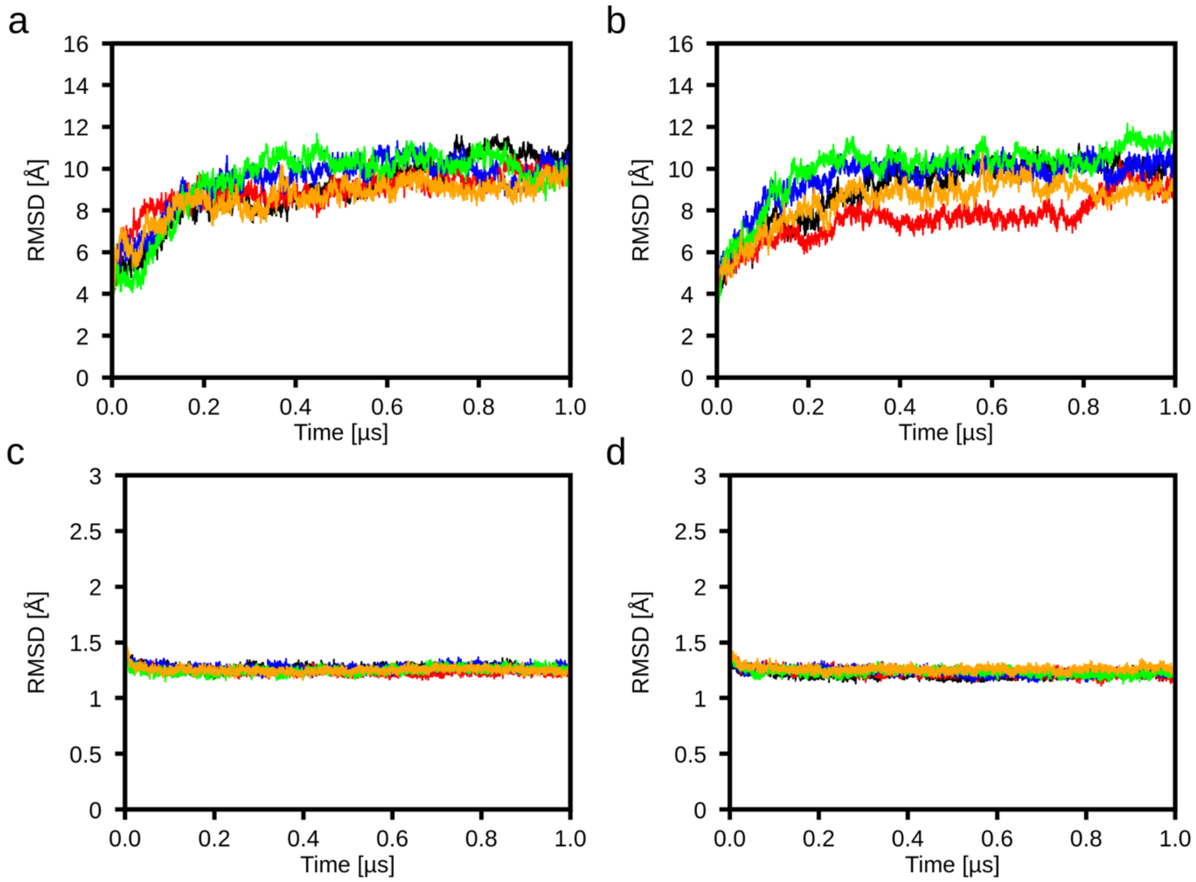

Supplementary Figure 12 | Conformational stability of the L1 fibril during molecular dynamics simulations.

Cα root mean square deviation (RMSD) relative to the cryo-EM structure of the L1 fibril throughout 1 µs MD simulations without restraints (**a**, **b**) and with additional weak restraints (0.1 kcal mol^{-1} Å^{-2}) on the Cα atoms (**c**, **d**). Each panel shows the RMSD data for five replica simulations (colored differently).

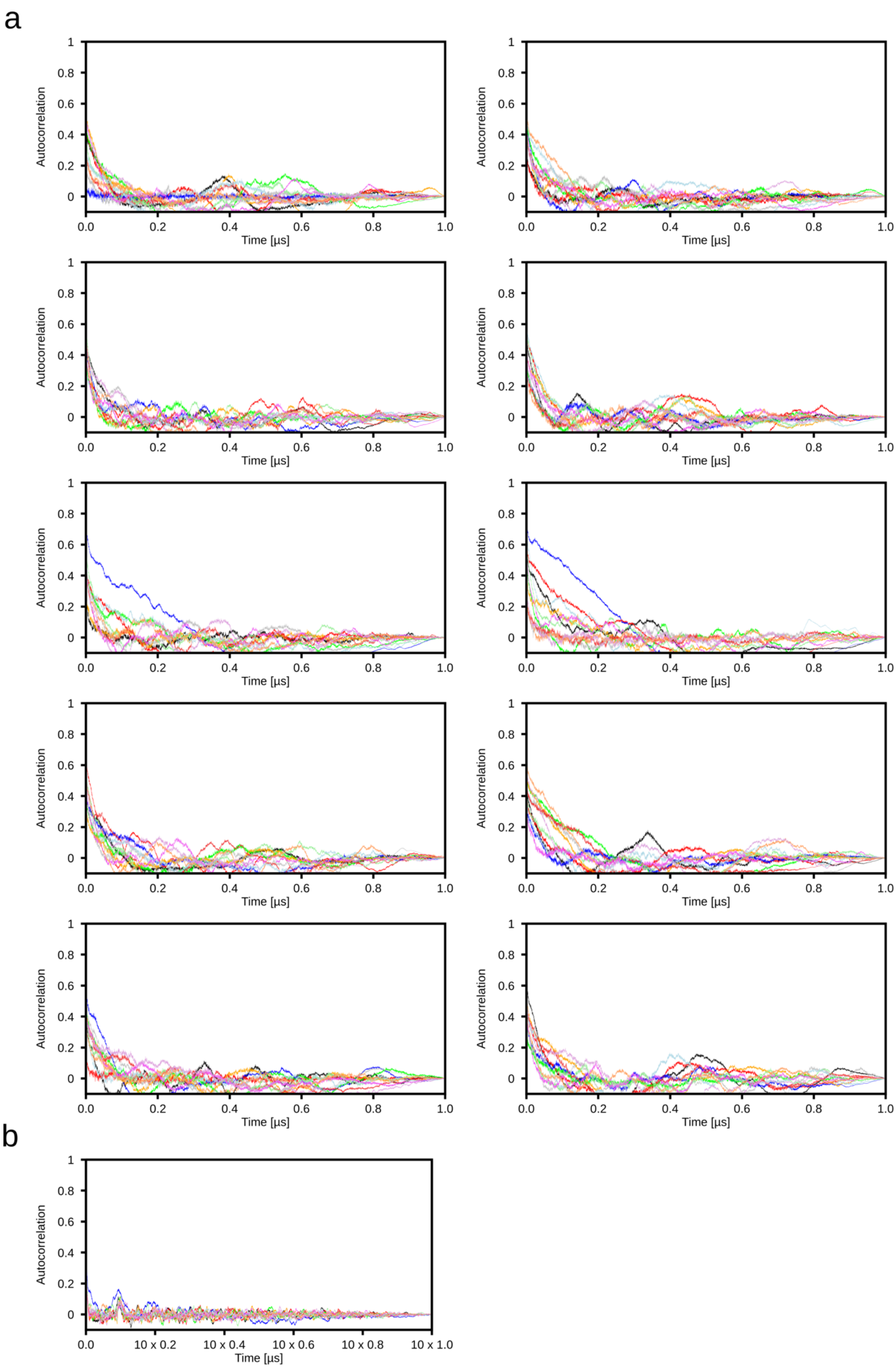

a
Autocorrelation
Time [µs]
0.0
0.2
0.4
0.6
0.8
1.0
0
0.2
0.4
0.6
0.8
1
b
10 x 0.2
10 x 0.4
10 x 0.6
10 x 0.8
10 x 1.0

Supplementary Figure 13 | Autocorrelation functions throughout molecular dynamics simulations the L1 fibril.

Autocorrelation functions of the hydrogen bond interactions between D23 and K28, for each replica simulation (n = 10) individually (**a**) and for the aggregated trajectory (10 x 1 μs) (**b**). Each line (colored differently) shows the autocorrelation function for one D23-K28 interaction pair. The autocorrelation analysis revealed major bumps within the first 400 ns and smaller bumps in during the 400 – 800 ns interval. After 800 ns, only a few individual interactions show minor fluctuations. Considering the aggregated trajectory, the bumps are smaller throughout the full simulation time, with only minor variations after 200 ns.

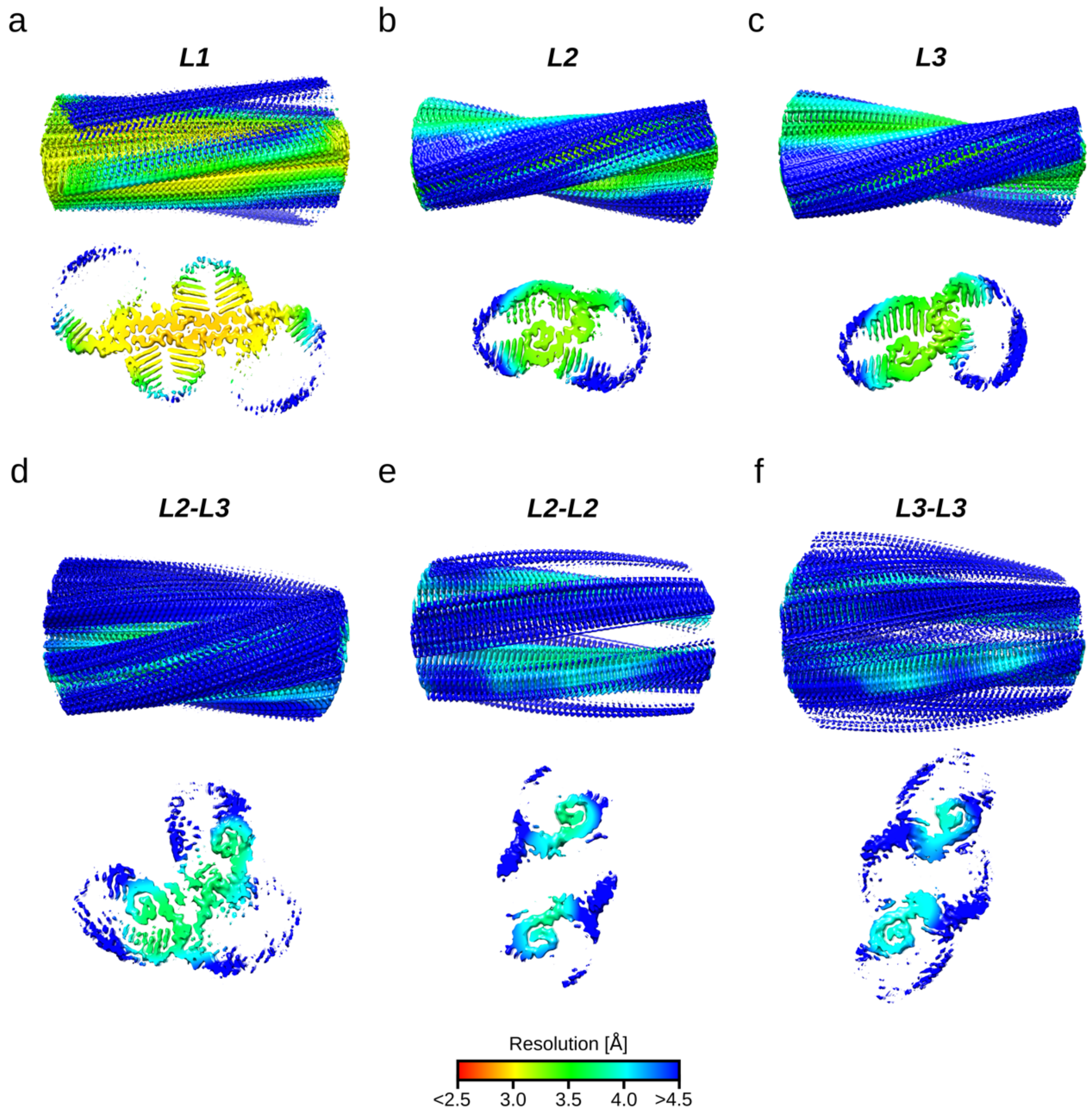

Supplementary Figure 14 | Local resolution estimation.

The sharpened and postprocessed maps of the *L1* (**a**), *L2* (**b**), *L3* (**c**), *L2-L3* (**d**), *L2-L2* (**e**), and *L3-L3* (**f**) fibrils colored according to the local resolution estimation (see color scale). The top panels show side-views of the maps, while the lower panels show cross-sections of the central region.

Supplementary Table 1 | Cryo-EM structure determination statistics.

Lipid-induced PM	*L1*	*L2*	*L3*	*L2-L3*	*L2-L2*	*L3-L3*
**Data collection**						
Microscope	Titan Krios G2					
Voltage [keV]	300					
Detector	K3					
Magnification	81,000					
Pixel size [Å]	1.05					
Defocus range [µm]	-0.7 to -2.0					
Exposure time [s/frame]	2.5					
Number of frames	40					
Total dose [e^-/Å^2]	~40.5 (~1.0 e^-/Å^2/frame)					
**Reconstruction**						
Micrographs	14,417					
Box width [pixels]	250					
Inter-box distance [pixels]	13					
Picked segments (no.)	3,384,825					
**Final map**						
PDB-ID	8ovk	8ovm	8owd	8owe	8owj	8owk
EMDB-ID	17218	17223	17234	17235	17238	17239
Final segments [no.]	177,981	83,126	51,100	13,034	15,185	19,050
Final resolution [Å] (FSC=0.143)	2.88	3.24	3.28	3.75	3.75	3.86
Applied map sharpening B-factor [Å^2]	-96.43	-96.96	-83.08	-91.50	-81.35	-105.93
Symmetry imposed	C1	C1	C1	C1	C1	C2
Helical rise [Å]	2.35	4.67	4.67	4.65	2.32	4.65
Helical twist [°]	179.63	-2.26	-2.07	-1.69	179.27	-1.52

Supplementary Table 2 | Model building statistics.

**Lipid-induced PM**	***L1***	***L2***	***L3***	***L2-L3***	***L2-L2***	***L3-L3***
**Initial model [PDB code]**	6w0o	*de novo*	*de novo*	*de novo*	*de novo*	*de novo*
**Model composition**						
Chains	10	5	5	10	10	10
Non-hydrogen atoms	3060	1360	1525	2940	2720	3050
Protein residues	400	180	200	385	360	400
**RMS deviations**						
Bond lengths [Å]	0.01	< 0.01	0.01	0.01	0.01	0.01
Bond angles [°]	1.80	0.99	2.44	2.47	2.26	3.09
**Validation**						
MolProbity score	2.42	1.67	2.18	2.52	1.82	2.05
Clashscore	4.35	9.68	5.03	11.09	7.26	14.24
**Ramachandran plot**						
Outliers [%]	0	0	0	0	0	0
Allowed [%]	5.26	2.94	0	1.37	0	0
Favored [%]	94.74	97.06	100	98.63	100	100

Supplementary Table 3 | ^{13}C, ^{15}N and ^{1}H chemical shifts of the lipidic Aβ40 fibril.

		H	N	C	CA	CB	CG	CD	CE	CZ	Nz
**3**	**E**	8.68	124.20	175.82	55.06	32.44	36.81	180.89			
**4**	**F**	8.14	113.63	176.12	60.81	45.25					
**5**	**R**	7.34	121.30	177.45	60.98	30.41	22.28	44.94		160.21	
**6**	**H**	8.28	120.96	173.95	51.99	32.56	132.54				
**7**	**D**	8.95	128.04	174.52	52.95	40.44	179.88				
**8**	**S**	9.04	116.58	176.47	56.38	66.98					
**9**	**G**	8.17	113.78	173.07	48.98						
**10**	**Y**	8.35	124.23	173.5	53.41	41.11	132.99			155.39	
**11**	**E**	8.17	121.81	173.89	55.29	33.15	35.67	181.90			
**12**	**V**	9.94	124.83	175.98	61.29						
**13**	**H**	8.25	120.96	171.72	51.95	33.87		118.09	137.87		
**14**	**H**	8.27	124.49	175.55	54.3	31.48	132.56	119.62	138.17		
**15**	**E**	8.23	122.46	173.79	54.97	51.88	34.09	179.14			
**16**	**K**	9.11	126.55	175.05	55.55	38.35	26.65	30.74	43.14		32.35
**17**	**L**	9.09	121.81	175.94	55.89	45.14	30.30				
**18**	**V**	8.71	122.31	174.38	61.71	36.23	22.28				
**19**	**F**	9.09	125.81	175.33	57.13	41.51	139.51		131.43		
**20**	**F**	9.16	124.79	175.31	57.99	43.24	138.82	132.90	132.65		
**21**	**A**	8.10	120.33	175.78	51.69	23.50					
**22**	**E**	8.27	120.76	174.13	55.03	33.70	36.46	183.37			
**23**	**D**	8.79	124.71	175.71	52.04	45.05	181.25				
**24**	**V**	9.09	120.53	176.02	60.74	37.88	25.06	22.26			
**25**	**G**	8.71	109.63	174.81	45.10						
**26**	**S**	8.51	118.73	173.73	60.16	68.07					
**27**	**N**	8.96	118.30	174.19	54.28	36.82	176.28				
**28**	**K**	8.03	114.47	175.97	55.03	42.09	28.81	30.37	45.46		34.99
**29**	**G**	7.94	102.61	172.67	45.77						
**30**	**A**	7.81	116.83	176.21	52.41	25.18					
**31**	**I**	7.88	120.40	175.42	61.69	41.46	17.84<br>28.77	14.74			
**32**	**I**	8.92	125.17	176.69	58.80	43.90	19.38<br>28.22	15.92			
**33**	**G**	8.56	114.54	172.43	49.88						
**34**	**L**	7.89	121.39	173.94	54.77	46.63	28.98	26.89			
**35**	**M**	9.35	127.14	174.77	55.24	39.15	33.04		23.30		
**36**	**V**	9.03	127.10	176.71	61.03	34.25	22.09				
**37**	**G**	9.03	112.37	172.61	46.67						
**39**	**V**	8.34	121.12		61.39	34.54					
**40**	**V**	9.24	124.90		62.18	36.58	23.97				
**23**	**D'**	8.82	124.58		55.55	42.14	179.7				
**28**	**K'**	8.88	129.19		55.83						

Exp	Spectrometer (MHz)	H-C-CP					C-N-CP					N-H-CP				H-N-CP				Sample
		tcp (ms)	H-rf (kHz)	shape	C-rf (kHz)	shape	tcp (ms)	C-rf (kHz)	shape	N-rf (kHz)	shape	tcp (ms)	N-rf (kHz)	shape	H-rf (kHz)	tcp (ms)	H-rf (kHz)	shape	N-rf (kHz)	
**(H)CANH**	800, Advance III	6	96	80-100%	102	square	19	163.5	tangent	64	square	0.55	95	100-80%	173					^{2}H,^{13}C,^{15}N Aβ40 fibril
**(H)CONH**	800, Advance III	7	96	90-100%	104	rectangle	19	163.5	tangent	65	rectangle	0.55	95	100-80%	173					^{2}H,^{13}C,^{15}N Aβ40 fibril
**(HCO)CA(CO)NH**	800, Advance III	7	96	80-100%	104	rectangle	19	163.5	tangent	65	rectangle	0.55	95	100-80%	173					^{2}H,^{13}C,^{15}N Aβ40 fibril
**(H)CO(CA)NH**	800, Advance III	7	96	90-100%	104	rectangle	19	163.5	tangent	64	square	0.55	95	100-80%	173					^{2}H,^{13}C,^{15}N Aβ40 fibril
**(HCA)CB(CA)NH**	800, Advance III	7	101	80-100%	101	rectangle	19	163.5	tangent	60	rectangle	0.55	98	100-80%	173					^{2}H,^{13}C,^{15}N Aβ40 fibril
**H(H)NH**	800, Advance III											0.45	97	100-80%	170	0.9	104	80-100%	170	^{2}H,^{13}C,^{15}N Aβ40 fibril
**(H)CANH**	800, Advance III	8	96	80-100%	88	square	20	139.1	tangent	57	square	0.9	85	100-80%	145					^{2}H,^{13}C,^{15}N Aβ40 fibril
**H(H)NHnoe**	800, Advance III											0.9	90	100-80%	152	0.9	99	80-100%	166	^{2}H,^{13}C,^{15}N Aβ40 fibril
**(H)CANH**	850, Advance III	7	98	85-100%	98	square	12	129	tangent	84	square	0.9	102	100-80%	170					^{2}H,^{13}C,^{15}N Aβ40 fibril
**(HCA)CB(CA)NH**	850, Advance III	7	98	85-100%	98	square	12	129	tangent	84	square	0.9	102	100-80%	170					^{2}H,^{13}C,^{15}N Aβ40 fibril
**(HCA)CB(CACO)NH**	850, Advance III	7	98	85-100%	98	square	18	129	tangent	84	square	0.9	102	100-80%	170					^{2}H,^{13}C,^{15}N Aβ40 fibril
**(H)CANH**	850, NEO	2.9	22	85-100%	17	square	25	17	tangent	15	square	0.85	20.7	100-80%	24					^{1}H,^{13}C,^{15}N Aβ40 fibril
**13C13C-DARR**	850, NEO	1.7	80		88	80-100%														^{1}H,^{13}C,^{15}N Aβ40 fibril
**(H)NCA**	850, NEO						3	16	100-90%	18	square					1.5	83	80-100%	73	^{1}H,^{13}C,^{15}N Aβ40 fibril

Supplementary Table 4 | Experimental parameters of NMR data acquisition on the sample.
