# Supplementary material for: Cryo-EM structures of lipidic fibrils of amyloid-β (1-40)
Source: Nat Commun. 2024 Feb 13;15:1297. doi: 10.1038/s41467-023-43822-x (PMC10864299; doi:10.1038/s41467-023-43822-x)
Supplement: Supplementary file 3 — Reporting Summary [file 41467_2023_43822_MOESM3_ESM.pdf]

nature portfolio

Corresponding author(s): G.F.Schröder and C.Griesinger

Last updated by author(s): Nov 18, 2023

Reporting Summary

Nature Portfolio wishes to improve the reproducibility of the work that we publish. This form provides structure for consistency and transparency in reporting. For further information on Nature Portfolio policies, see our Editorial Policies and the Editorial Policy Checklist.

Statistics

For all statistical analyses, confirm that the following items are present in the figure legend, table legend, main text, or Methods section.

n/a	Confirmed	
☐	☒	The exact sample size (*n*) for each experimental group/condition, given as a discrete number and unit of measurement
☐	☒	A statement on whether measurements were taken from distinct samples or whether the same sample was measured repeatedly
☒	☐	The statistical test(s) used AND whether they are one- or two-sided<br>*Only common tests should be described solely by name; describe more complex techniques in the Methods section.*
☒	☐	A description of all covariates tested
☒	☐	A description of any assumptions or corrections, such as tests of normality and adjustment for multiple comparisons
☐	☒	A full description of the statistical parameters including central tendency (e.g. means) or other basic estimates (e.g. regression coefficient) AND variation (e.g. standard deviation) or associated estimates of uncertainty (e.g. confidence intervals)
☒	☐	For null hypothesis testing, the test statistic (e.g. *F*, *t*, *r*) with confidence intervals, effect sizes, degrees of freedom and *P* value noted<br>*Give P values as exact values whenever suitable.*
☒	☐	For Bayesian analysis, information on the choice of priors and Markov chain Monte Carlo settings
☒	☐	For hierarchical and complex designs, identification of the appropriate level for tests and full reporting of outcomes
☒	☐	Estimates of effect sizes (e.g. Cohen's *d*, Pearson's *r*), indicating how they were calculated

Our web collection on statistics for biologists contains articles on many of the points above.

Software and code

Policy information about availability of computer code

Data collection	Bruker TopSpin(V3.6.5 and V4), SerialEM(V4.0)
Data analysis	NMR data : CcpNMR(V 2.4.2), TopSpin(V 3.6.5)<br>Cryo-EM data : RELION(V3.1), CTFFIND(V4.1), COOT(V0.9), PHENIX(V1.19)<br>MD simulation : Amber(V22)<br>Chimera(V1.8)

For manuscripts utilizing custom algorithms or software that are central to the research but not yet described in published literature, software must be made available to editors and reviewers. We strongly encourage code deposition in a community repository (e.g. GitHub). See the Nature Portfolio guidelines for submitting code & software for further information.

Data

Policy information about availability of data

All manuscripts must include a data availability statement. This statement should provide the following information, where applicable:

- Accession codes, unique identifiers, or web links for publicly available datasets
- A description of any restrictions on data availability
- For clinical datasets or third party data, please ensure that the statement adheres to our policy

NMR spectra raw data generated in this study for the assigned chemical shift data (HN, Cα, Cβ, and C′) of Amyloid beta (1-40) fibril were deposited in the BMRB

under the accession number 52006 (https://doi.org/10.13018/BMR52006). Cyro-EM maps have been deposited in the Electron Microscopy Data bank (EMDB) under the accession numbers EMD-17218(L1) https://www.ebi.ac.uk/pdbe/entry/emdb/EMD-17218, EMD-17223(L2) https://www.ebi.ac.uk/pdbe/entry/emdb/EMD-17223, EMD-17234(L3) https://www.ebi.ac.uk/pdbe/entry/emdb/EMD-17234, EMD-17235(L2-L3) https://www.ebi.ac.uk/pdbe/entry/emdb/EMD-17235, EMD-17238(L2-L2) https://www.ebi.ac.uk/pdbe/entry/emdb/EMD-17238, and EMD-17239(L3-L3) https://www.ebi.ac.uk/pdbe/entry/emdb/EMD-17239. The corresponding atomic models have been deposited in the Protein Data Bank (PDB) under the accession number 8ovk (L1)(DOI: https://doi.org/10.2210/pdb8OVK/pdb), 8ovm (L2)(DOI: https://doi.org/10.2210/pdb8OVM/pdb), 8owd (L3)(DOI: https://doi.org/10.2210/pdb8OWD/pdb), 8owe (L2-L3) (https://doi.org/10.2210/pdb8OWE/pdb), 8owj (L2-L2)(DOI: https://doi.org/10.2210/pdb8OWJ/pdb), and 8owk (L3-L3)(DOI: https://doi.org/10.2210/pdb8OWK/pdb).

Research involving human participants, their data, or biological material

Policy information about studies with human participants or human data. See also policy information about sex, gender (identity/presentation), and sexual orientation and race, ethnicity and racism.

Reporting on sex and gender	Not applicable
Reporting on race, ethnicity, or other socially relevant groupings	Not applicable
Population characteristics	Not applicable
Recruitment	Not applicable
Ethics oversight	Not applicable

Note that full information on the approval of the study protocol must also be provided in the manuscript.

Field-specific reporting

Please select the one below that is the best fit for your research. If you are not sure, read the appropriate sections before making your selection.

- [x] Life sciences
- [ ] Behavioural & social sciences
- [ ] Ecological, evolutionary & environmental sciences

For a reference copy of the document with all sections, see nature.com/documents/nr-reporting-summary-flat.pdf

Life sciences study design

All studies must disclose on these points even when the disclosure is negative.

Sample size	Fibril preparation: The fibril sample was checked by Negative staining EM, and the structural reproducibility was checked by NMR experiments (hCANH, hNCA) from different labeled samples (1H13C15N, 2H13C,15N). NMR experiments: The number of repetitions was adapted to the signal-to-noise obtained. Measurements were continued until sufficient signal-to-noise was obtained. For Cryo-EM, we collected one dataset, including 14,417 micrographs. NMR, Cryo-EM, and Negative staining EM were measured on the 1H13C15N fibril sample.
Data exclusions	For NMR exp: No data were excluded.<br>Standard image classification procedures(Scheres, J. Struc. Biol. 180:519-530, (2021)) were employed to select particle images to reach the highest resolution reconstructions. Details of the number of selected images are given in Supplementary Table S1.
Replication	During fibril formation, the sample is cross-checked by Thioflavin T Fluorescence, CD spectrum (Every 12h), and NMR experiment (hCANH, hNH). Four times repeated the same method for different NMR experiments and observed the same spectrum on the (hCANH, hNH). The Cyro-EM sample was cross-checked by the same method (Tht, CD, Negative staining EM, and NMR). For the MD simulation of the L1 fibril, we performed ten replica simulations.
Randomization	Randomization was not performed for the both methods.
Blinding	No blinding on the NMR exp. Adjustment of NMR parameters requires prior knowledge of isotope labeling. The long measurement times (days to weeks) will be multiplied if the parameters are incorrect. Blinding was not performed on cyro-EM because the risk of bias in the results was considered negligible.

Reporting for specific materials, systems and methods

We require information from authors about some types of materials, experimental systems and methods used in many studies. Here, indicate whether each material, system or method listed is relevant to your study. If you are not sure if a list item applies to your research, read the appropriate section before selecting a response.

Materials & experimental systems

n/a	Involved in the study
☒	☐ Antibodies
☒	☐ Eukaryotic cell lines
☒	☐ Palaeontology and archaeology
☒	☐ Animals and other organisms
☒	☐ Clinical data
☒	☐ Dual use research of concern
☒	☐ Plants

Methods

n/a	Involved in the study
☒	☐ ChIP-seq
☒	☐ Flow cytometry
☒	☐ MRI-based neuroimaging

Plants

Seed stocks	Not applicable
Novel plant genotypes	Not applicable
Authentication	Not applicable
